# Aberrant orbitofrontal cortical activation interferes with encoding of Pavlovian fear conditioning

**DOI:** 10.3389/fnbeh.2022.981041

**Published:** 2022-08-22

**Authors:** Chung-Fu Sun, Chun-hui Chang

**Affiliations:** ^1^Institute of Systems Neuroscience, National Tsing Hua University, Hsinchu, Taiwan; ^2^Brain Research Center, National Tsing Hua University, Hsinchu, Taiwan

**Keywords:** fear conditioning, fear circuitry, orbitofrontal cortex, mental disorder, rat

## Abstract

Obsessive-compulsive disorder (OCD) patients were usually found with the hyper-activation of the orbitofrontal cortex (OFC) and a deficit in fear extinction learning. The OFC can be subdivided into the lateral OFC (lOFC) and the medial OFC (mOFC). Previous studies have suggested that both subregions are involved in the modulation of negative emotions. However, how aberrant activation of the OFC interacts with the encoding of Pavlovian fear remains unknown. In this study, the lOFC or the mOFC was pharmacologically activated or inactivated before the fear conditioning on Day 1, followed by a context test on Day 2 and a tone test on Day 3 in male Long-Evans rats. We found that for the animals that underwent fear conditioning under aberrant activation of either the lOFC or the mOFC, they showed normal within-session fear expression. However, the acquisition/consolidation of contextual fear was impaired under mOFC activation, while the acquisition/consolidation of cued fear was impaired under either the lOFC or the mOFC activation, in that these animals showed lower freezing compared to controls during the retrieval test. On the other hand, for the animals that underwent fear conditioning under inactivation of either the lOFC or the mOFC, they showed normal within-session fear expression, as well as intact encoding of both the contextual and cued fear. Together, our results suggested that the OFC was not actively engaged in the acquisition of Pavlovian fear conditioning, but aberrant activation of the OFC impaired fear learning.

## Introduction

Fear, an innate defensive mechanism rooted in animals, is essential for survival (Adolphs, 2013; LoBue et al., 2019). In laboratory settings, Pavlovian fear conditioning is frequently used to investigate the fear-based neurobiology of learning and memory (Kim and Jung, 2006; Sun et al., 2020). After repeated pairings of the neutral conditioned stimulus (CS, e.g., a tone) and unconditioned stimulus (US, e.g., a foot shock), the CS becomes capable to evoke the fear response (e.g., freezing) (Domjan, 2005). The conditioned fear is also associated with the general context where the conditioning occurred, in that the fear response can be triggered when the animals are reintroduced into the conditioned context (Fanselow, 2000; Curzon et al., 2009).

Several brain areas are involved in fear regulation, including the amygdala, the medial prefrontal cortex (mPFC), and the hippocampus (HPC) (LeDoux, 2000; Shin and Liberzon, 2010; Tovote et al., 2015). The inputs of the CS and the US converge in the lateral amygdala (LA) (LeDoux, 2000; Campese et al., 2015; Holmes et al., 2022), with the contextual information reaching the basal amygdala (BA) through the HPC (Antoniadis and McDonald, 2000; LeDoux, 2000). The processed signals from the LA and the BA pass on to the central amygdala (CeA), which is the output interface that regulates fear expression (LeDoux, 2000). Two subregions of the mPFC, the infralimbic (IL) and the prelimbic (PL) cortices, also serve as crucial moderators of fear regulation (Giustino et al., 2016). Inputs from the IL through the intercalated cell (ITC) (Li et al., 2011; Hagihara et al., 2021) are instrumental in decreasing fear output from CeA, while the PL is associated with the high fear state (Tovote et al., 2015). The HPC is mostly involved in the contextual fear conditioning, for example, the encoding of the environmental cues during the conditioning process (Fanselow, 2000).

Exposure to adverse factors like stress is one of the reasons leading to the development of mental disorders, such as anxiety-related disorders, post-traumatic stress disorder (PTSD), and obsessive-compulsive disorder (OCD) (Gonzalez and Martinez, 2014; Steimer, 2022). Patients with stress-related mental disorders often experienced depression, phobia, and anhedonia (Shalev, 2009; Hamm, 2020; Taschereau-Dumouchel et al., 2022). Evidence from neuroimaging studies has revealed the involvement of the orbitofrontal cortex (OFC) in psychiatric disorders (Jackowski et al., 2012). The OFC can be subdivided into the lateral OFC (lOFC) and medial OFC (mOFC) (McDonald et al., 1996; Hampshire et al., 2012). The lOFC is associated with negative emotions and obsessions, while the mOFC is related to decision-making, especially the response to rewards (Milad and Rauch, 2007; Rushworth et al., 2011; Noonan et al., 2017). The OFC could interact with the fear circuit through its extensive connections with the mPFC (Vertes, 2004; Sul et al., 2010; Murphy and Deutch, 2018) and the amygdala (Barreiros et al., 2021). Clinically, the aberrant OFC activities interfere with the acquisition and retention of fear extinction (Ursu and Carter, 2009; Lagemann et al., 2012). Some earlier studies have suggested that the OFC response was enhanced in PTSD patients (Thomaes et al., 2013) and the OFC was involved in the functional brain networks of OCD (Bijanki et al., 2021). In addition, other studies also revealed that PTSD and OCD patients failed to sustain the suppression of extinguished fear (Milad et al., 2013; Garfinkel et al., 2014).

Previously, we have demonstrated that pharmacological activation of the lOFC or the mOFC impaired the acquisition of fear extinction in rats (Chang et al., 2018; Hsieh and Chang, 2020). Moreover, lOFC activation during extinction resulted in the failure of context-dependent retrieval of extinction memory (Shih and Chang, 2021). These results supported the clinical findings that aberrant OFC activities weakened the efficacy of exposure-based extinction therapies. However, OFC hyper-activation is a chronic condition in OCD patients (Maia et al., 2008; Robbins et al., 2019), and how such condition interacts with the acquisition of conditioned fear remains unknown.

In this study, the lOFC and the mOFC were pharmacologically activated or inactivated immediately before the Pavlovian fear conditioning (Day 1), followed by the context test (Day 2) and tone test (Day 3). We hypothesized that pre-conditioning OFC activation may interfere with the fear encoding due to its robust connection with the fear circuit, while pre-conditioning OFC inactivation may leave the fear encoding intact because the OFC is not required for the association of the CS and the US. These hypotheses were tested under weak fear conditioning procedure (two trials), leaving room for the animals to show increase or decrease of fear expression.

## Materials and methods

### Subjects

A total of 174 male Long-Evans rats (National Laboratory Animal Center, Taiwan), aged 6–8 weeks upon arrival, were housed in individual cages in a temperature (22 ± 1°C) and humidity (60–70%) controlled facility at National Yang Ming Chiao Tung University with a 12-h light/dark cycle (lights on at 7 a.m.) and *ad libitum* access to food and water. Each rat was handled once a day for at least 1 week before the surgery. All the procedures were approved by the Institutional Animal Care and Use Committees of National Tsing Hua University and National Yang Ming Chiao Tung University.

### Surgery

The animals were anesthetized with ketamine (80–100 mg/kg) and xylazine (8–10 mg/kg) for the surgery. Core body temperature was maintained at approximately 37°C by the feedback monitor throughout the entire procedure, and the rat was fixed in a stereotaxic instrument. In Experiment 1 and 2, two stainless steel guide cannulae (26-gauge, Plastics One) were implanted bilaterally in all rats aiming at the lOFC [relative to bregma, anteroposterior (AP) +3.5 mm, mediolateral (ML) ±3.0 mm, and dorsoventral (DV) −4.0 mm]. In Experiment 3 and 4, a single guide cannula aiming at the mOFC was implanted in all rats [relative to bregma, (AP) +4.4 mm, (ML) +1.4 mm, and (DV) −4.4 mm] with 15° angle toward the midline (entering side counterbalanced). Three additional anchor screws were mounted and the headstage was fixed in place with dental acrylic. Carprofen (5 mg/kg) was subcutaneously administered immediately after the surgery followed by intraperitoneal injections for two additional days to reduce pain. All animals were placed back in their home cages and monitored until awake. The rats were allowed a recovery period of 4–7 days before the start of the behavioral procedures, during which the dummies (Plastics One) that extended 1.0 mm beyond the guide cannulae were changed daily to avoid cannula obstruction.

### Behavioral apparatus

Four fear conditioning chambers (Med-Associates, VT, United States) were used with two context settings. In context A, animals were transported to the chambers in transparent cuboids. The chamber lights were on, and the doors of the chambers were half-open. The fans in the chambers were in operation, which also worked as background noise. The pans beneath the chambers were filled with 1% acetic acid to provide a distinct odor. In context B, animals were transported to the chambers in cylinders and covered with black sheets. The lights in the behavioral room were blurred red, which was a dark surrounding for rats. The chamber lights were off, and the doors of the chambers were closed. The fans in the chambers were off. The pans were filled with 1% ammonium. Additionally, there were A-frame inserts and acrylic plates above the grids inside the chambers.

### Behavioral procedures

**FIGURE 1:**
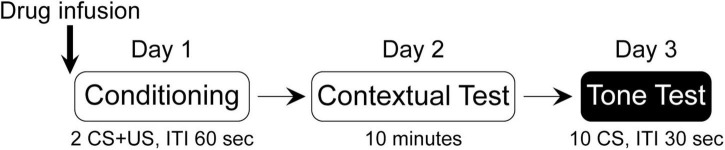
Experimental design of the study.

A mixed design was conducted with the between-subject factors of “Group” (conditioning “Cond” and no-conditioning “NoCond”) and “Drug” [vehicle “V” and N-methyl-D-aspartate (NMDA) “N”/muscimol “M”], and the within-subject factor of “Trial” (or “Minute”), yielding a total of four groups [V-Cond, V-NoCond, N(M)-Cond, and N(M)-NoCond] in each experiment. Drug infusion was performed immediately before the acquisition of the fear conditioning session (Figure 1). On Day 1, the Cond rats were presented with two trials of co-terminated tone (10 s, 2 kHz, 85 dB)-footshock (2 s, 1.0 mA) pairings with inter-stimulus intervals (ISIs) of 60 s, while the NoCond rats were presented with tones only. On Day 2, all the rats were placed back into the conditioning context (Context A) for 10 min to assess the contextual fear. On Day 3, all the rats were tested with 10 trials of tones alone in a shifted context (Context B) with ISIs of 30 s. For each squad, four rats entered the chambers, with the groups and orders counterbalanced. Previously, we have shown that there was no difference of innate fear to the context settings (Shih and Chang, 2021), and the physical contexts of context A and B were not counterbalanced. All the experiments were conducted during the light phase between 7 a.m. and 7 p.m.

### Drug infusion

N-methyl-D-aspartate (0.75 μg/0.5 μl; Experiment 1 and 3), GABA_*A*_ agonist “muscimol” (0.5 μg/0.5 μl; Experiment 2 and 4), or saline (0.5 μl) was infused at the rate of 0.2 μl/min using injectors (33-gauge, Plastics One) extended 1.0 mm beyond the guide cannulae. NMDA and muscimol were used to locally activate or inactive the targeted brain areas, respectively. The injectors were attached to plastic tubes, which were connected to Hamilton syringes and an infusion pump. After the infusion, one extra minute was allowed for drug infusion before the injectors were retrieved. The NMDA dosage was determined based on previous reports (Chang et al., 2018; Hsieh and Chang, 2020). Early studies (Legault et al., 2000; Floresco et al., 2001) have demonstrated that the physiological effects of NMDA at this dosage last up to about 2–3 h, which outlast our fear conditioning sessions. The muscimol dosage was determined based on previous reports (Ramanathan et al., 2018). The effects of muscimol last approximately 3 h after the injection (van Duuren et al., 2007). Therefore, the drugs interfered with both the acquisition and early consolidation phase of fear encoding.

### Histology

**FIGURE 2:**
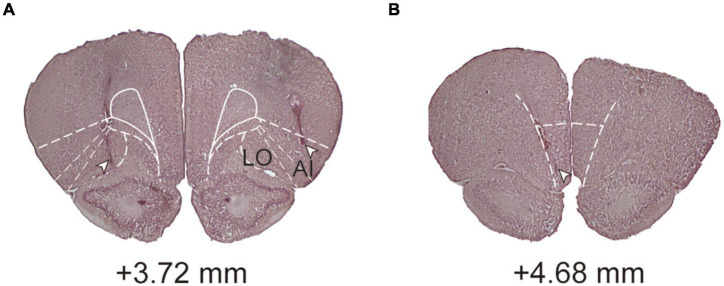
Histological analyses and conformation. **(A)** A coronal section showing an example of cannula placement in the lOFC. **(B)** A coronal section showing an example of cannula placement in the mOFC. Level of +3.72 and +4.68 mm, anterior relative to bregma. LO, lateral orbital area; AI, agranular insula.

After the conclusion of all the behavioral procedures, the rats were euthanized by CO_2_ asphyxiation, decapitated, and with their brains removed. The brains were fixed in 8% paraformaldehyde (PFA) in 0.2 M phosphate buffer (PB) for 3–5 days and then were transferred into 25% sucrose in 0.1 M PB until saturation. Afterward, 60-μm coronal sections were obtained on a cryostat at −20°C, mounted onto subbed slides, and stained with the standard Nissl stain after drying to confirm cannula implantation (Figure 2). The lateral orbital (LO) area and adjacent agranular insula (AI; including the ventral and dorsal subdivisions) were counted as the lOFC (McDonald et al., 1996; Rempel-Clower, 2007). Animals with cannula tips located outside the lOFC or mOFC were excluded from behavioral analysis.

### Behavioral analysis

The behavior of every rat was recorded using Video Freeze (Med-Associates, VT, United States; motion threshold at 100, sampling at 0.2 s), and immobility was defined as consecutively observed movements for 1 s below the threshold. The immobility during the 3-min pre-CS period was considered the baseline (BL). Furthermore, immobility during the ISIs (conditioning and tone test) or for every minute (context test) was reported. The percentage of total observations in which immobility occurred was calculated and these values were submitted to repeated measures of analysis of variance (RM ANOVA). After significant F ratios (*p* < 0.05) were obtained, Student-Newman-Keuls (S-N-K) *post-hoc* analysis was performed if necessary. For all ANOVAs, effect sizes were reported as partial eta square (η*_*p*_*^2^) (Fritz et al., 2012; Lakens, 2013). As an effect size, η*_*p*_*^2^ values 0.01, 0.06, and 0.14 are generally interpreted as a small, medium, and large effects, respectively (Richardson, 2011). All statistical analyses (Richardson, 2011) were performed using SPSS (IBM), and all data are presented as mean ± SEM.

## Results

### Experiment 1: Fear encoding under lateral OFC activation

In this experiment, the lOFC was pharmacologically activated during conditioning to assess how aberrant lOFC activities interfere with fear encoding.

#### Histology and final groups

**FIGURE 3:**
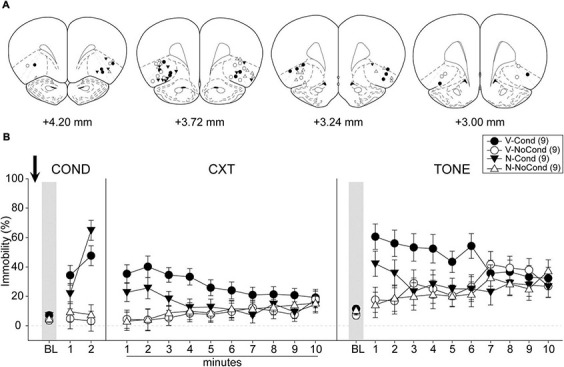
Lateral OFC (lOFC) activation during fear conditioning interfered with the acquisition/consolidation of cued fear. **(A)** Injection sites in the lOFC for all the animals included in data analyses at levels of +4.20, +3.72,+3.24, and +3.00 mm, anterior relative to bregma. **(B)** Percentage of immobility during the conditioning (left panel), contextual test (middle panel), and tone test (right panel). N, N-methyl-D-aspartate (NMDA); V, vehicle; Cond, conditioning; NoCond, no-conditioning. All data are shown as the mean ± SEM.

A total of 48 rats were used in Experiment 1. One rat was excluded from analyses due to death during surgery before the start of the behavioral procedures and 11 rats were excluded due to cannula misplacements. All cannula placements of the animals included in data analyses in this experiment are shown in Figure 3A. The final group sizes were: V-Cond (*n* = 9), V-NoCond (*n* = 9), N-Cond (*n* = 9), and N-NoCond (*n* = 9).

#### Behavioral results

On Day 1, immediately after the drug infusion of saline or NMDA, the animals were placed into the chambers for fear conditioning procedures (Figure 3B, left panel). Both the Cond groups showed an overall increase in immobility as the trials progressed, while the NoCond groups exhibited low immobility throughout the conditioning session. There were significant main effects of “Group” [*F*(1, 32) = 41.00, *p* < 0.001, η*_*p*_*^2^ = 0.56] and “Trial” [*F*(2, 64) = 25.07, *p* < 0.001, η*_*p*_*^2^ = 0.44], and a significant two-way interaction between “Group” and “Trial” [*F*(2, 64) = 23.15, *p* < 0.001, η*_*p*_*^2^ = 0.42]. The lack of any “Drug” main effect or interactions suggested the equivalent in-session fear expression of the Cond groups.

On Day 2, the animals were placed into the conditioning context to assess their contextual fear (Figure 3B, middle panel). Both the Cond groups expressed higher immobility initially, while the NoCond groups demonstrated low immobility. There was a significant main effect of “Group” [*F*(1, 32) = 8.13, *p* = 0.008, η*_*p*_*^2^ = 0.20] and a significant two-way interaction between “Group” and “Minute” [*F*(9, 288) = 6.01, *p* < 0.001, η*_*p*_*^2^ = 0.16]. The lack of any “Drug” main effect or interactions suggested that lOFC activation before the fear conditioning did not interfere with the acquisition/consolidation of contextual fear. The significant “Group” and “Minute” interaction indicated that the Cond animals started to show extinction to the context toward latter of the session.

On Day 3, the animals were placed into a novel context to assess their cued fear (Figure 3B, right panel). The V-Cond group showed higher immobility than the N-Cond group, and both the Cond groups exhibited higher immobility than the NoCond groups at initial trials. There were significant main effects of “Group” [*F*(1, 32) = 4.43, *p* < 0.05, η*_*p*_*^2^ = 0.12] and “Trial” [*F*(10, 320) = 4.11, *p* < 0.001, η*_*p*_*^2^ = 0.11], a marginal main effect of “Drug” [*F*(1, 32) = 3.84, *p* = 0.059, η*_*p*_*^2^ = 0.11], and a significant two-way interaction between “Group” and “Trial” [*F*(10, 320) = 4.15, *p* < 0.001, η*_*p*_*^2^ = 0.12]. The marginal “Drug” main effect suggested a trend that pre-conditioning lOFC activation impaired the acquisition/consolidation of cued fear. The significant “Group” and “Trial” interaction indicated that the Cond animals started to show extinction to the tones toward latter trials.

### Experiment 2: Fear encoding under lateral OFC inactivation

The results of Experiment 1 revealed that there was a trend of impaired acquisition of cued fear when the lOFC was activated during the fear conditioning. To determine whether the lOFC was actively engaged in the fear encoding, we inactivated the lOFC before the conditioning in this experiment.

#### Histology and final groups

**FIGURE 4:**
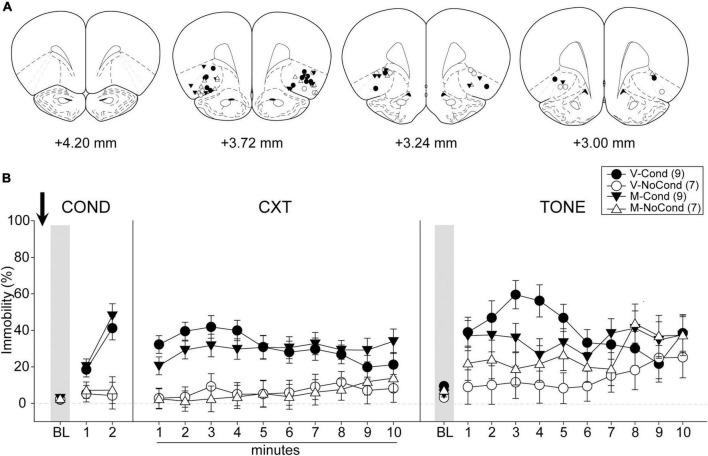
Lateral OFC (lOFC) inactivation during fear conditioning left the acquisition/consolidation of fear intact. **(A)** Injection sites in the lOFC for all the animals included in data analyses at levels of +4.20, +3.72, +3.24, and +3.00 mm, anterior relative to bregma. **(B)** Percentage of immobility during the conditioning (left panel), contextual test (middle panel), and tone test (right panel). M, muscimol; V, vehicle; Cond, conditioning; NoCond, no-conditioning. All data are shown as the mean ± SEM.

A total of 42 rats were used in Experiment 2. Five rats were excluded due to cannula misplacements, and five rats were excluded due to lesions induced by contamination of guide cannulae. All cannula placements of the animals included in data analyses in this experiment are shown in Figure 4A. The final group sizes were: V-Cond (*n* = 9), V-NoCond (*n* = 7), N-Cond (*n* = 9), and N-NoCond (*n* = 7).

#### Behavioral results

On Day 1, immediately after the drug infusion of saline or muscimol, the animals were placed into the chambers for fear conditioning procedures (Figure 4B, left panel). Both the Cond groups showed an overall increase in immobility as the trials progressed, while the NoCond groups exhibited low immobility throughout the conditioning session. There were significant main effects of “Group” [*F*(1, 28) = 25.42, *p* < 0.001, η*_*p*_*^2^ = 0.48] and “Trial” [*F*(2, 56) = 32.92, *p* < 0.001, η*_*p*_*^2^ = 0.54], and a significant two-way interaction between “Group” and “Trial” [*F*(2, 56) = 24.74, *p* < 0.001, η*_*p*_*^2^ = 0.47]. The lack of any “Drug” main effect or interactions suggested equivalent in-session fear expression of the Cond groups.

On Day 2, the animals were placed into the conditioning context to assess their contextual fear (Figure 4B, middle panel). Both the Cond groups expressed high immobility, while the NoCond groups demonstrated low immobility. There was a significant main effect of “Group” [*F*(1, 28) = 31.05, *p* < 0.001, η*_*p*_*^2^ = 0.53]. The lack of any “Drug” main effect or interactions suggested that lOFC inactivation before the fear conditioning did not interfere with the acquisition/consolidation of contextual fear.

On Day 3, the animals were placed into a novel context to assess their cued fear (Figure 4B, right panel). Both the Cond groups exhibited higher immobility than the NoCond groups at initial trials. There were significant main effects of “Group” [*F*(1, 28) = 7.10, *p* = 0.01, η*_*p*_*^2^ = 0.20] and “Trial” [*F*(10, 280) = 4.91, *p* < 0.001, η*_*p*_*^2^ = 0.15], and a significant two-way interaction between “Group” and “Trial” [*F*(10, 280) = 2.69, *p* = 0.004, η*_*p*_*^2^ = 0.09]. The lack of any “Drug” main effect or interactions suggested that lOFC inactivation before the fear conditioning did not interfere with the acquisition/consolidation of cued fear. The significant “Group” and “Trial” interaction indicated that the Cond animals started to show extinction to the tones toward latter trials.

### Experiment 3: Fear encoding under medial OFC activation

The results of Experiment 1 and 2 revealed that the lOFC was not actively engaged in fear encoding, but there was a trend that aberrant activation of the lOFC during conditioning impaired the encoding of cued fear. We further investigated the role of the mOFC on fear encoding in Experiment 3 and 4. We first examined the fear encoding under mOFC activation in this experiment.

#### Histology and final groups

**FIGURE 5:**
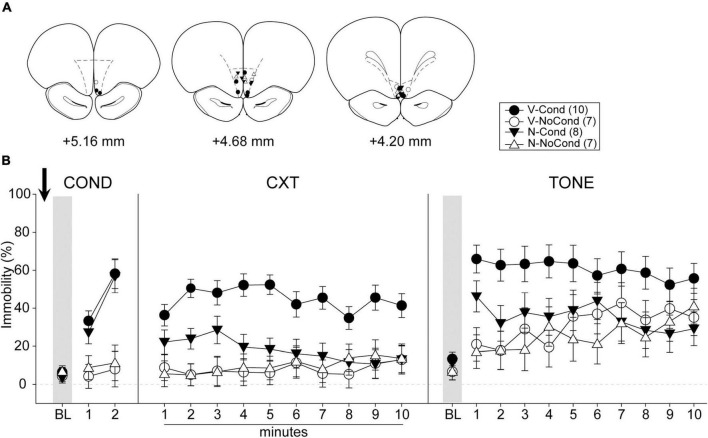
Medial OFC (mOFC) activation during fear conditioning interfered with the acquisition/consolidation of contextual fear and cued fear. **(A)** Injection sites in the mOFC for all the animals included in data analyses at levels of +5.16, +4.68, and +4.20 mm, anterior relative to bregma. **(B)** Percentage of immobility during the conditioning (left panel), contextual test (middle panel), and tone test (right panel). N, N-methyl-D-aspartate (NMDA); V, vehicle; Cond, conditioning; NoCond, no-conditioning. All data shown as the mean ± SEM.

A total of 48 rats were used in Experiment 3. Two rats were excluded from analyses due to death during surgeries before the start of the behavioral procedures, and 14 rats were excluded due to cannula misplacements. Five animals (V-NoCond, *n* = 2; N-NoCond, *n* = 3) in the NoCond groups underwent the no-conditioning procedure without tone presentations due to the protocol setting errors. All cannula placements of the animals included in data analyses in this experiment are shown in Figure 5A. The final group sizes were: V-Cond (*n* = 10), V-NoCond (*n* = 7), N-Cond (*n* = 8), and N-NoCond (*n* = 7).

#### Behavioral results

On Day 1, immediately after the drug infusion of saline or NMDA, the animals were placed into the chambers for fear conditioning procedures (Figure 5B, left panel). Both the Cond groups showed an overall increase in immobility as the trials progressed, while the NoCond groups exhibited low immobility throughout the conditioning session. There were significant main effects of “Group” [*F*(1, 28) = 25.18, *p* < 0.001, η*_*p*_*^2^ = 0.47] and “Trial” [*F*(2, 56) = 29.49, *p* < 0.001, η*_*p*_*^2^ = 0.51], and a significant two-way interaction between “Group” and “Trial” [*F*(2, 56) = 21.66, *p* < 0.001, η*_*p*_*^2^ = 0.44]. The lack of any “Drug” main effect or interactions suggested the equivalent in-session fear expression of the Cond groups.

On Day 2, the animals were placed into the conditioning context to assess their contextual fear (Figure 5B, middle panel). The V-Cond group showed higher immobility than the N-Cond group, and both the Cond groups exhibited higher immobility than the NoCond groups. There were significant main effects of “Group” [*F*(1, 28) = 20.71, *p* < 0.001, η*_*p*_*^2^ = 0.43] and “Drug” [*F*(1, 28) = 6.48, *p* = 0.017, η*_*p*_*^2^ = 0.19], and significant two-way interactions between “Group” and “Drug” [*F*(1, 28) = 8.49, *p* = 0.007, η*_*p*_*^2^ = 0.23] and between “Group” and “Minute” [*F*(9, 252) = 2.22, *p* = 0.02, η*_*p*_*^2^ = 0.07]. The significant “Group” and “Drug” interaction suggested that pre-conditioning mOFC activation impaired the acquisition/consolidation of contextual fear in the Cond groups. Moreover, the significant “Group” and “Minute” interaction indicated that the Cond animals started to show extinction to the context toward latter of the session.

On Day 3, the animals were placed into a novel context to assess their cued fear (Figure 5B, right panel). The V-Cond group showed higher immobility than the N-Cond group, and both the Cond groups exhibited higher immobility than the NoCond groups at initial trials. There were significant main effects of “Group” [*F*(1, 28) = 7.25, *p* = 0.01, η*_*p*_*^2^ = 0.21], “Drug” [*F*(1, 28) = 4.35, *p* = 0.046, η*_*p*_*^2^ = 0.13], and “Trial” [*F*(10, 280) = 6.72, *p* < 0.001, η*_*p*_*^2^ = 0.19], and a significant two-way interaction between “Group” and “Trial” [*F*(10, 280) = 2.70, *p* = 0.004, η*_*p*_*^2^ = 0.09]. The significant “Drug” main effect suggested that pre-conditioning mOFC activation also impaired the acquisition/consolidation of cued fear. Moreover, the significant “Group” and “Trial” interaction indicated that the Cond animals started to show extinction to the tones toward latter trials.

### Experiment 4: Fear encoding under medial OFC inactivation

The results of Experiment 3 revealed that the acquisition of both the contextual and cued fear were impaired when the mOFC was activated during the fear conditioning. To determine whether the mOFC was actively engaged in fear encoding, we inactivated the mOFC before conditioning in this experiment.

#### Histology and final groups

**FIGURE 6:**
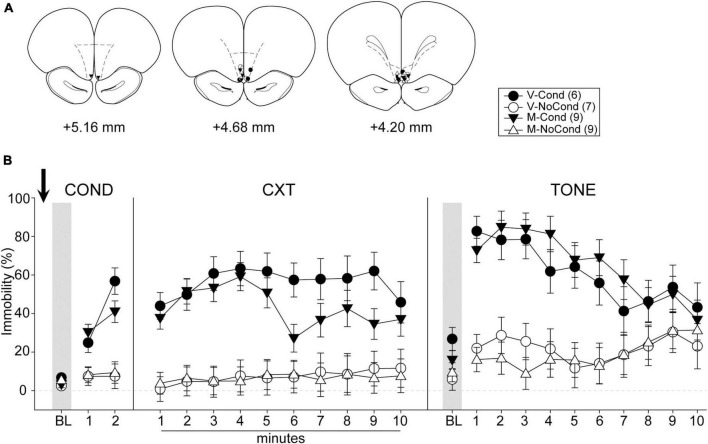
Medial OFC (mOFC) inactivation during fear conditioning left the acquisition/consolidation of fear intact. **(A)** Injection sites in the mOFC for all the animals included in data analyses at levels of +5.16, +4.68, and +4.20 mm, anterior relative to bregma. **(B)** Percentage of immobility during the conditioning (left panel), contextual test (middle panel), and tone test (right panel). M, muscimol; V, vehicle; Cond, conditioning; NoCond, no-conditioning. All data are shown as the mean ± SEM.

A total of 36 rats were used in Experiment 4. Five rats were excluded due to cannula misplacements. All cannula placements of the animals included in data analyses in this experiment are shown in Figure 6A. The final group sizes were: V-Cond (*n* = 6), V-NoCond (*n* = 7), N-Cond (*n* = 9), and N-NoCond (*n* = 9).

#### Behavioral results

On Day 1, immediately after the drug infusion of saline or muscimol, the animals were placed into the chambers for fear conditioning procedures (Figure 6B, left panel). Both the Cond groups showed an overall increase in immobility as the trials progressed, while the NoCond groups exhibited low immobility throughout the conditioning session. There were significant main effects of “Group” [*F*(1, 27) = 37.31, *p* < 0.001, η*_*p*_*^2^ = 0.58] and “Trial” [*F*(2, 54) = 41.88, *p* < 0.001, η*_*p*_*^2^ = 0.64], and a significant two-way interaction between “Group” and “Trial” [*F*(2, 54) = 27.96, *p* < 0.001, η*_*p*_*^2^ = 0.51]. The lack of any “Drug” main effect or interactions suggested equivalent in-session fear expression of the Cond groups.

On Day 2, the animals were placed into the conditioning context to assess their contextual fear (Figure 6B, middle panel). Both the Cond groups expressed high immobility, while the NoCond groups demonstrated low immobility. There were significant main effects of “Group” [*F*(1, 27) = 36.94, *p* < 0.001, η*_*p*_*^2^ = 0.58] and “Minute” [*F*(9, 243) = 2.10, *p* = 0.03, η*_*p*_*^2^ = 0.07], and a significant two-way interaction between “Group” and “Minute” [*F*(9, 243) = 2.31, *p* = 0.02, η*_*p*_*^2^ = 0.08]. The lack of any “Drug” main effect or interactions suggested that mOFC inactivation before fear conditioning did not interfere with the acquisition/consolidation of contextual fear. The significant “Group” and “Minute” interaction indicated that the Cond animals started to show extinction to the context toward latter of the session.

On Day 3, the animals were placed into a novel context to assess their cued fear (Figure 6B, right panel). Both the Cond groups exhibited higher immobility than the NoCond groups. There were significant main effects of “Group” [*F*(1, 27) = 41.31, *p* < 0.001, η*_*p*_*^2^ = 0.61] and “Trial” [*F*(10, 270) = 7.40, *p* < 0.001, η*_*p*_*^2^ = 0.22], and a significant two-way interaction between “Group” and “Trial” [*F*(10, 270) = 6.65, *p* < 0.001, η*_*p*_*^2^ = 0.20]. The lack of any “Drug” main effect or interactions suggested that mOFC inactivation before fear conditioning did not interfere with the acquisition/consolidation of cued fear. The significant “Group” and “Trial” interaction indicated that the Cond animals started to show extinction to the tones toward latter trials.

## Discussion

In this study, we examined how the animals encoded Pavlovian contextual and cued fear under aberrant OFC activation or inactivation. Our data revealed that there was a trend of impaired encoding of cued fear when the lOFC was activated during the acquisition/consolidation phase (Experiment 1). On the other hand, mOFC activation before conditioning impaired the encoding of both the contextual and cued fear (Experiment 3). However, inactivation of the lOFC (Experiment 2) or the mOFC (Experiment 4) left the learning intact. Together, our results support our hypotheses that aberrant OFC activation interfered with the encoding of Pavlovian fear conditioning, but OFC inactivation would not. Moreover, the CS-US association was weakened under aberrant OFC activation during fear conditioning.

Hyper-activation of the OFC is a chronic condition in OCD patients (Maia et al., 2008; Robbins et al., 2019). Earlier studies have shown that when the OFC was hyperactive, the acquisition of fear extinction and suppression of extinguished fear were impaired in clinical and animal studies (Milad et al., 2013; Chang et al., 2018; Hsieh and Chang, 2020). Nonetheless, how aberrant OFC activation interacts with the process of new fear learning was not addressed. Because of the extensive connection of the OFC with the fear circuit, especially the mPFC and the amygdala (Gabbott et al., 2005; Hoover and Vertes, 2007, 2011), we reasoned that hyperactive OFC would impact the encoding of conditioned fear as well. Due to the uncertainty of whether the association between the CS and the US would be strengthened or weakened under OFC activation, a weak training procedure with only two trials was used in this study, followed by assessments of the contextual and cued fear. Pharmacological activation of either the lOFC or the mOFC did not interfere with in-session fear expression during conditioning. Our data revealed that there were down-shifts in contextual fear and cued fear when assessed the following 2 days after conditioning in both experiments (Experiment 1 and 3). While mOFC activation during conditioning significantly impaired the encoding of both the contextual and cued fear (Experiment 3), lOFC activation resulted in a trend of impaired encoding only of the cued fear (Experiment 1). Statistically, we concluded that aberrant lOFC activation did not interfere with the encoding of contextual fear. However, it is likely that the weak conditioning procedure masked the effect due to the low contextual fear in the control group (V-Cond), and the impairment due to lOFC activation may emerge if the rats were conditioned under a strong training procedure with more trials.

The different functions of the lOFC and the mOFC may have contributed to the disparate results in this study as well. Earlier studies have suggested distinct roles of the lOFC and the mOFC in negative emotion learning and expressing, in that the lOFC is important for the fear regulation (Ray et al., 2018), while the mOFC is implicated in the fear extinction (Milad and Rauch, 2007). The mOFC may have a more potent role in the encoding phase of learning and the consequent recall. Indeed, aberrant activation of the OFC, especially the mOFC, impaired both the fear encoding and fear extinction acquisition, suggesting that the impairments we observed in this study may be a more general learning deficit. On the other hand, inactivation of the lOFC or the mOFC during the encoding of fear left the learning process intact (Experiment 2 and 4). Together, the results suggested that the OFC was not actively engaged in the acquisition/consolidation of conditioned fear under normal circumstances, consistent with earlier studies (Ma et al., 2020).

We noticed some limitations in this study. First of all, only male rats were used in the study, and therefore the results should be interpreted carefully in the absence of the female rat data. Several rodent and human studies have pointed out the sex difference in the regulation and expression of emotions (McLean and Anderson, 2009; Cover et al., 2014). For example, men tend to have richer emotional experience, while women have higher emotional expressivity (Deng et al., 2016). In rodents, the sex difference in the fear extinction and renewal were reported (Binette et al., 2022). Another limitation is that the mOFC was activated and inactivated unilaterally. Unilateral mOFC activation was sufficient to generate the learning deficits (Experiment 3), but we could not rule out the possibility that the intact side of the mOFC was sufficient to maintain normal function under unilateral inactivation (Experiment 4). Bilateral mOFC inactivation needs to be further examined to address this issue. Finally, there is the likelihood that lOFC or mOFC activation/inactivation may have effects on locomotor activities *per se*. However, the retrieval of contextual fear (Day 2) and cued fear (Day 3) was assessed drug free in all the experiments, and therefore the decreased immobility (Experiment 1 and 3) more likely reflected the learning deficits.

The weakened fear learning under OFC hyper-activation may not be a good thing for the survival purpose of the animals or the proper function of human beings. The inappropriate encoding between the environmental and discrete cues with aversive stimuli may result in a blunted sense of danger. Our results provided some insights that through the control of the abnormal activities of the OFC in patients with some psychiatric disorders, their symptoms may be alleviated to restore the proper function for daily life.

## Data availability statement

The raw data supporting the conclusions of this article will be made available by the authors, without undue reservation.

## Ethics statement

The animal study was reviewed and approved by the Institutional Animal Care and Use Committees of National Tsing Hua University and National Yang Ming Chiao Tung University.

## Author contributions

C-hC designed the experiments. C-FS performed the experiments and analyzed the data. C-FS and C-hC wrote the manuscript. Both authors have read and approved the final manuscript.

## References

[B1] Adolphs R. (2013). The biology of fear. *Curr. Biol.* 23 R79–R93. 10.1016/j.cub.2012.11.055 23347946 PMC3595162

[B2] Antoniadis E. A. McDonald R. J. (2000). Amygdala, hippocampus and discriminative fear conditioning to context. *Behav. Brain Res.* 108 1–19. 10.1016/S0166-4328(99)00121-7 10680753

[B3] Barreiros I. V. Panayi M. C. Walton M. E. (2021). Organization of Afferents along the Anterior-posterior and Medial-lateral Axes of the Rat Orbitofrontal Cortex. *Neuroscience* 460 53–68. 10.1016/j.neuroscience.2021.02.017 33609638 PMC8022030

[B4] Bijanki K. R. Pathak Y. J. Najera R. A. Storch E. A. Goodman W. K. Simpson H. B. (2021). Defining functional brain networks underlying obsessive-compulsive disorder (OCD) using treatment-induced neuroimaging changes: a systematic review of the literature. *J. Neurol. Neurosurg. Psychiatry* 92 776–786. 10.1136/jnnp-2020-324478 33906936 PMC8223624

[B5] Binette A. N. Totty M. S. Maren S. (2022). Sex differences in the immediate extinction deficit and renewal of extinguished fear in rats. *PLoS One* 17:e0264797. 10.1371/journal.pone.0264797 35687598 PMC9187087

[B6] Campese V. D. Gonzaga R. Moscarello J. M. Ledoux J. E. (2015). Modulation of instrumental responding by a conditioned threat stimulus requires lateral and central amygdala. *Front. Behav. Neurosci.* 9:293. 10.3389/fnbeh.2015.00293 26578921 PMC4626560

[B7] Chang Y. H. Liu S. W. Chang C. H. (2018). Pharmacological activation of the lateral orbitofrontal cortex on regulation of learned fear and extinction. *Neurobiol. Learn. Mem.* 148 30–37. 10.1016/j.nlm.2017.12.011 29294382

[B8] Cover K. K. Maeng L. Y. Lebron-Milad K. Milad M. R. (2014). Mechanisms of estradiol in fear circuitry: implications for sex differences in psychopathology. *Transl. Psychiatry* 4:e422. 10.1038/tp.2014.67 25093600 PMC4150242

[B9] Curzon P. Rustay N. R. Browman K. E. (2009). “Cued and Contextual Fear Conditioning for Rodents,” in *Methods of Behavior Analysis in Neuroscience*, ed. Buccafusco J. J. (Boca Raton, FL: CRC Press). 10.1201/NOE1420052343.ch2 21204331

[B10] Deng Y. Chang L. Yang M. Huo M. Zhou R. (2016). Gender Differences in Emotional Response: Inconsistency between Experience and Expressivity. *PLoS One* 11 e0158666. 10.1371/journal.pone.0158666 27362361 PMC4928818

[B11] Domjan M. (2005). Pavlovian conditioning: a functional perspective. *Annu. Rev. Psychol.* 56 179–206. 10.1146/annurev.psych.55.090902.141409 15709933

[B12] Fanselow M. S. (2000). Contextual fear, gestalt memories, and the hippocampus. *Behav. Brain Res.* 110 73–81. 10.1016/S0166-4328(99)00186-2 10802305

[B13] Floresco S. B. Todd C. L. Grace A. A. (2001). Glutamatergic afferents from the hippocampus to the nucleus accumbens regulate activity of ventral tegmental area dopamine neurons. *J. Neurosci.* 21 4915–4922. 10.1523/JNEUROSCI.21-13-04915.2001 11425919 PMC6762358

[B14] Fritz C. O. Morris P. E. Richler J. J. (2012). Effect size estimates: current use, calculations, and interpretation. *J. Exp. Psychol. Gen.* 141 2–18. 10.1037/a0024338 21823805

[B15] Gabbott P. L. Warner T. A. Jays P. R. Salway P. Busby S. J. (2005). Prefrontal cortex in the rat: projections to subcortical autonomic, motor, and limbic centers. *J. Comp. Neurol.* 492 145–177. 10.1002/cne.20738 16196030

[B16] Garfinkel S. N. Abelson J. L. King A. P. Sripada R. K. Wang X. Gaines L. M. (2014). Impaired contextual modulation of memories in PTSD: an Fmri and psychophysiological study of extinction retention and fear renewal. *J. Neurosci.* 34 13435–13443. 10.1523/JNEUROSCI.4287-13.2014 25274821 PMC4262698

[B17] Giustino T. F. Fitzgerald P. J. Maren S. (2016). Fear Expression Suppresses Medial Prefrontal Cortical Firing in Rats. *PLoS One* 11:e0165256. 10.1371/journal.pone.0165256 27776157 PMC5077087

[B18] Gonzalez P. Martinez K. G. (2014). The role of stress and fear in the development of mental disorders. *Psychiatr. Clin. North Am.* 37 535–546. 10.1016/j.psc.2014.08.010 25455064 PMC4255725

[B19] Hagihara K. M. Bukalo O. Zeller M. Aksoy-Aksel A. Karalis N. Limoges A. (2021). Intercalated amygdala clusters orchestrate a switch in fear state. *Nature* 594 403–407. 10.1038/s41586-021-03593-1 34040259 PMC8402941

[B20] Hamm A. O. (2020). Fear, anxiety, and their disorders from the perspective of psychophysiology. *Psychophysiology* 57:e13474. 10.1111/psyp.13474 31529522

[B21] Hampshire A. Chaudhry A. M. Owen A. M. Roberts A. C. (2012). Dissociable roles for lateral orbitofrontal cortex and lateral prefrontal cortex during preference driven reversal learning. *Neuroimage* 59 4102–4112. 10.1016/j.neuroimage.2011.10.072 22075266 PMC3391678

[B22] Holmes N. M. Fam J. P. Clemens K. J. Laurent V. Westbrook R. F. (2022). The neural substrates of higher-order conditioning: A review. *Neurosci. Biobehav. Rev.* 138:104687. 10.1016/j.neubiorev.2022.104687 35561894

[B23] Hoover W. B. Vertes R. P. (2007). Anatomical analysis of afferent projections to the medial prefrontal cortex in the rat. *Brain Struct. Funct.* 212 149–179. 10.1007/s00429-007-0150-4 17717690

[B24] Hoover W. B. Vertes R. P. (2011). Projections of the medial orbital and ventral orbital cortex in the rat. *J. Comp. Neurol.* 519 3766–3801. 10.1002/cne.22733 21800317

[B25] Hsieh H. T. Chang C. H. (2020). Activation of medial orbitofrontal cortex abolishes fear extinction and interferes with fear expression in rats. *Neurobiol. Learn. Mem.* 169:107170. 10.1016/j.nlm.2020.107170 31978551

[B26] Jackowski A. P. Araujo Filho G. M. Almeida A. G. Araujo C. M. Reis M. Nery F. (2012). The involvement of the orbitofrontal cortex in psychiatric disorders: an update of neuroimaging findings. *Braz. J. Psychiatry* 34 207–212. 10.1590/S1516-44462012000200014 22729418

[B27] Kim J. J. Jung M. W. (2006). Neural circuits and mechanisms involved in Pavlovian fear conditioning: a critical review. *Neurosci. Biobehav. Rev.* 30 188–202. 10.1016/j.neubiorev.2005.06.005 16120461 PMC4342048

[B28] Lagemann T. Rentzsch J. Montag C. Gallinat J. Jockers-Scherubl M. Winter C. (2012). Early orbitofrontal hyperactivation in obsessive-compulsive disorder. *Psychiatry Res.* 202 257–263. 10.1016/j.pscychresns.2011.10.002 22809741

[B29] Lakens D. (2013). Calculating and reporting effect sizes to facilitate cumulative science: a practical primer for t-tests and ANOVAS. *Front. Psychol.* 4:863. 10.3389/fpsyg.2013.00863 24324449 PMC3840331

[B30] LeDoux J. E. (2000). Emotion circuits in the brain. *Annu. Rev. Neurosci.* 23 155–184. 10.1146/annurev.neuro.23.1.155 10845062

[B31] Legault M. Rompre P. P. Wise R. A. (2000). Chemical stimulation of the ventral hippocampus elevates nucleus accumbens dopamine by activating dopaminergic neurons of the ventral tegmental area. *J. Neurosci.* 20 1635–1642. 10.1523/JNEUROSCI.20-04-01635.2000 10662853 PMC6772387

[B32] Li G. Amano T. Pare D. Nair S. S. (2011). Impact of infralimbic inputs on intercalated amygdala neurons: a biophysical modeling study. *Learn. Mem.* 18 226–240. 10.1101/lm.1938011 21436395 PMC3072774

[B33] LoBue V. Kim E. Delgado M. (2019). Fear in infancy: Lessons from snakes, spiders, heights, and strangers. *Devel. Psychol.* 55 1889–1907. 10.1037/dev0000675 31464493 PMC6716607

[B34] Ma C. Jean-Richard-Dit-Bressel P. Roughley S. Vissel B. Balleine B. W. Killcross S. (2020). Medial orbitofrontal cortex regulates instrumental conditioned punishment, but not pavlovian conditioned fear. *Cereb Cortex Commun.* 1:tgaa039. 10.1093/texcom/tgaa039 34296108 PMC8152850

[B35] Maia T. V. Cooney R. E. Peterson B. S. (2008). The neural bases of obsessive-compulsive disorder in children and adults. *Dev. Psychopathol.* 20 1251–1283. 10.1017/S0954579408000606 18838041 PMC3079445

[B36] McDonald A. J. Mascagni F. Guo L. (1996). Projections of the medial and lateral prefrontal cortices to the amygdala: a Phaseolus vulgaris leucoagglutinin study in the rat. *Neuroscience* 71 55–75. 10.1016/0306-4522(95)00417-3 8834392

[B37] McLean C. P. Anderson E. R. (2009). Brave men and timid women? A review of the gender differences in fear and anxiety. *Clin. Psychol. Rev.* 29 496–505. 10.1016/j.cpr.2009.05.003 19541399

[B38] Milad M. R. Furtak S. C. Greenberg J. L. Keshaviah A. Im J. J. Falkenstein M. J. (2013). Deficits in conditioned fear extinction in obsessive-compulsive disorder and neurobiological changes in the fear circuit. *JAMA Psychiatry* 70 608–618. 10.1001/jamapsychiatry.2013.914 23740049

[B39] Milad M. R. Rauch S. L. (2007). The role of the orbitofrontal cortex in anxiety disorders. *Ann. N Y Acad. Sci.* 1121 546–561. 10.1196/annals.1401.006 17698998

[B40] Murphy M. J. M. Deutch A. Y. (2018). Organization of afferents to the orbitofrontal cortex in the rat. *J. Comp. Neurol.* 526 1498–1526. 10.1002/cne.24424 29524205 PMC5899655

[B41] Noonan M. P. Chau B. K. H. Rushworth M. F. S. Fellows L. K. (2017). Contrasting Effects of Medial and Lateral Orbitofrontal Cortex Lesions on Credit Assignment and Decision-Making in Humans. *J. Neurosci.* 37 7023–7035. 10.1523/JNEUROSCI.0692-17.2017 28630257 PMC6705719

[B42] Ramanathan K. R. Ressler R. L. Jin J. Maren S. (2018). Nucleus Reuniens Is Required for Encoding and Retrieving Precise, Hippocampal-Dependent Contextual Fear Memories in Rats. *J. Neurosci.* 38 9925–9933. 10.1523/JNEUROSCI.1429-18.2018 30282726 PMC6234294

[B43] Ray M. H. Hanlon E. Mcdannald M. A. (2018). Lateral orbitofrontal cortex partitions mechanisms for fear regulation and alcohol consumption. *PLoS One* 13:e0198043. 10.1371/journal.pone.0198043 29856796 PMC5983516

[B44] Rempel-Clower N. L. (2007). Role of orbitofrontal cortex connections in emotion. *Ann. N. Y. Acad. Sci.* 1121 72–86. 10.1196/annals.1401.026 17846152

[B45] Richardson J. T. E. (2011). Eta squared and partial eta squared as measures of effect size in educational research. *Educ. Res. Rev.* 6 135–147. 10.1016/j.edurev.2010.12.001

[B46] Robbins T. W. Vaghi M. M. Banca P. (2019). Obsessive-Compulsive Disorder: Puzzles and Prospects. *Neuron* 102 27–47. 10.1016/j.neuron.2019.01.046 30946823

[B47] Rushworth M. F. Noonan M. P. Boorman E. D. Walton M. E. Behrens T. E. (2011). Frontal cortex and reward-guided learning and decision-making. *Neuron* 70 1054–1069. 10.1016/j.neuron.2011.05.014 21689594

[B48] Shalev A. Y. (2009). Posttraumatic stress disorder and stress-related disorders. *Psychiatr. Clin. North Am.* 32 687–704. 10.1016/j.psc.2009.06.001 19716997 PMC2746940

[B49] Shih C. W. Chang C. H. (2021). Medial or lateral orbitofrontal cortex activation during fear extinction differentially regulates fear renewal. *Behav. Brain Res.* 412:113412. 10.1016/j.bbr.2021.113412 34118296

[B50] Shin L. M. Liberzon I. (2010). The neurocircuitry of fear, stress, and anxiety disorders. *Neuropsychopharmacology* 35 169–191. 10.1038/npp.2009.83 19625997 PMC3055419

[B51] Steimer T. (2022). The biology of fear- and anxiety-related behaviors. *Dialog. Clin. Neurosci.* 4 231–249. 10.31887/DCNS.2002.4.3/tsteimer PMC3181681 22033741

[B52] Sul J. H. Kim H. Huh N. Lee D. Jung M. W. (2010). Distinct roles of rodent orbitofrontal and medial prefrontal cortex in decision making. *Neuron* 66 449–460. 10.1016/j.neuron.2010.03.033 20471357 PMC2872629

[B53] Sun Y. Gooch H. Sah P. (2020). Fear conditioning and the basolateral amygdala. *F1000Res* 9:F1000FacultyRev–53. 10.12688/f1000research.21201.1 32047613 PMC6993823

[B54] Taschereau-Dumouchel V. Michel M. Lau H. Hofmann S. G. Ledoux J. E. (2022). Putting the “mental” back in “mental disorders”: a perspective from research on fear and anxiety. *Mol. Psychiatry* 27 1322–1330. 10.1038/s41380-021-01395-5 35079126 PMC9095479

[B55] Thomaes K. Dorrepaal E. Draijer N. De Ruiter M. B. Elzinga B. M. Sjoerds Z. (2013). Increased anterior cingulate cortex and hippocampus activation in Complex Ptsd during encoding of negative words. *Soc. Cogn. Affect. Neurosci.* 8 190–200. 10.1093/scan/nsr084 22156722 PMC3575721

[B56] Tovote P. Fadok J. P. Luthi A. (2015). Neuronal circuits for fear and anxiety. *Nat. Rev. Neurosci.* 16 317–331. 10.1038/nrn3945 25991441

[B57] Ursu S. Carter C. S. (2009). An initial investigation of the orbitofrontal cortex hyperactivity in obsessive-compulsive disorder: exaggerated representations of anticipated aversive events? *Neuropsychologia* 47 2145–2148. 10.1016/j.neuropsychologia.2009.03.018 19467363 PMC2688401

[B58] van Duuren E. Van Der Plasse G. Van Der Blom R. Joosten R. N. Mulder A. B. Pennartz C. M. (2007). Pharmacological manipulation of neuronal ensemble activity by reverse microdialysis in freely moving rats: a comparative study of the effects of tetrodotoxin, lidocaine, and muscimol. *J. Pharmacol. Exp. Ther.* 323 61–69. 10.1124/jpet.107.124784 17626795

[B59] Vertes R. P. (2004). Differential projections of the infralimbic and prelimbic cortex in the rat. *Synapse* 51 32–58. 10.1002/syn.10279 14579424

